# High prevalence of hepatitis C virus infection and low level of awareness among people who recently started injecting drugs in a cross-sectional study in Germany, 2011–2014: missed opportunities for hepatitis C testing

**DOI:** 10.1186/s12954-019-0338-y

**Published:** 2020-01-10

**Authors:** Julia Enkelmann, Martyna Gassowski, Stine Nielsen, Benjamin Wenz, Stefan Roß, Ulrich Marcus, Viviane Bremer, Ruth Zimmermann, DRUCK Study group, Claus-Thomas Bock, Norbert Bannert, Claudia Santos-Hövener, Norbert Scherbaum, Jürgen Klee, Andreas Hecht, Dirk Schaeffer

**Affiliations:** 1 Postgraduate Training for Applied Epidemiology, Robert Koch Institute, Berlin, Germany; 2 European Programme for Intervention Epidemiology Training, ECDC, Stockholm, Sweden; 3 Department of Infectious Disease Epidemiology, Robert Koch Institute, Berlin, Germany; 4 Department of Infectious Disease Epidemiology, Division for HIV/AIDS, STI and Blood-borne Infections, Robert Koch Institute, Berlin, Germany; 5 Charité University Medicine, Berlin, Germany; 6 Institute of Virology, National Reference Centre for Hepatitis C, University Hospital Essen, University of Duisburg-Essen, Essen, Germany

**Keywords:** HCV, PWID, New injectors, Hepatitis C testing, Germany

## Abstract

**Background:**

In Germany, risk of hepatitis C virus (HCV) infection is highest among people who inject drugs (PWID). New injectors (NI) are particularly vulnerable for HCV-acquisition, but little is known about health seeking behaviour and opportunities for intervention in this group. We describe characteristics, HCV prevalence, estimated HCV incidence and awareness of HCV-status among NIs and missed opportunities for hepatitis C testing.

**Methods:**

People who had injected drugs in the last 12 months were recruited into a cross-sectional serobehavioural study using respondent-driven sampling in 8 German cities, 2011–2014. Data on sociodemographic characteristics, previous HCV testing and access to care were collected through questionnaire-based interviews. Capillary blood was tested for HCV. People injecting drugs < 5 years were considered NI.

**Results:**

Of 2059 participants with available information on duration of injection drug use, 232 (11% were NI. Estimated HCV incidence among NI was 19.6 infections/100 person years at risk (95% CI 16–24). Thirty-six percent of NI were HCV-positive (thereof 76% with detectable RNA) and 41% of those HCV-positive were unaware of their HCV-status. Overall, 27% of NI reported never having been HCV-tested. Of NI with available information, more than 80% had attended low-threshold drug services in the last 30 days, 24% were released from prison in the last 12 months and medical care was most commonly accessed in hospitals, opioid substitution therapy (OST)-practices, practices without OST and prison hospitals.

**Conclusion:**

We found high HCV-positivity and low HCV-status awareness among NI, often with missed opportunities for HCV-testing. To increase early diagnosis and facilitate treatment, HCV-testing should be offered in all facilities, where NI can be reached, especially low-threshold drug services and addiction therapy, but also prisons, hospitals and practices without OST.

## Background

Chronic hepatitis C virus (HCV) infection can lead to liver cirrhosis, liver failure and hepatocellular carcinoma. Currently, no effective vaccine exists but infections can be cured with antiviral treatment. The WHO aims at eliminating viral hepatitis as a public health threat by 2030 [1] and Germany has committed to this elimination agenda. A joint strategy for HIV, hepatitis B/C and other sexually transmitted infections was published by the German Ministry of Health in 2016 [2]. Major obstacles to overcome include a high proportion of people who are not aware of their infection and, linkage to care [3].

Germany is a low prevalence country for HCV infection. In a population-based survey of the general adult population living in Germany conducted in 2008–2011, HCV-antibody prevalence was 0.3% and HCV-RNA prevalence 0.2% [4]. People who inject drugs (PWID) are underrepresented in this survey and account for nearly 80% of newly diagnosed HCV infections notified in Germany with information on the mode of transmission [5].

Several studies have found HCV incidence to be highest in the first years of injection drug use (IDU) [6, 7], but little is known about the health seeking behaviour and opportunities for intervention in people who recently began injecting drugs, which in the following are referred to as “new injectors” (NI). Therefore, we analysed data from a cross-sectional study among PWID in Germany to describe HCV prevalence, estimated incidence and missed opportunities for HCV-testing and promotion of prevention measures in this group, with a focus on settings that could be used to reach NI in Germany and similar countries.

## Methods

We analysed data from the DRUCK-study, a cross-sectional study conducted between 2011 and 2014 using respondent-driven sampling to recruit PWID that had injected drugs in the last 12 months in one of eight German cities (Berlin, Essen, Leipzig, Munich, Frankfurt, Hanover, Hamburg, Cologne). Data on sociodemographic characteristics, previous HCV testing and access to care were collected through questionnaire-based face-to-face interviews. Capillary blood was tested for HCV antibodies and RNA. More detailed methods and the full study protocol have been published elsewhere [8, 9]. To capture all participants who had been exposed to HCV, we defined participants with detectable HCV antibody and/or HCV-RNA as HCV-positive for this analysis.

We defined NI as people injecting drugs for less than 5 years and long-term injectors (LI) as people injecting drugs for 5 years or longer.

Stata version 15.1 was used to carry out statistical analyses. *X*^2^-tests were performed and odds ratios using univariable logistic regression were calculated to compare groups.

Assuming that all participants were HCV-negative before they began injecting drugs, we estimated HCV incidence among NI as follows: date of study participation, month and year of birth and age when IDU was initiated was collected. Using stochastic simulation and assuming uniform distribution, we simulated the (unknown) month injection drug use was initiated and the (unknown) later time point HCV infection occurred based on 200 realisations in each case. For each realisation, we performed a bootstrap to account for the sampling error and characterised the resulting probability distribution by its mean and the 2.5 and 97.5 percentiles.

## Results

Of 2077 participants that provided a blood sample, information on duration of IDU was available for 2059 of whom 232 (11%) were NI (range 8.1% in Cologne (former West Germany) - 19.8% in Leipzig (former East Germany)).

Of NI, 31% were female, 27% were first-generation migrants and 22% reported being homeless (defined as reporting living on the streets or in homeless shelters as main residence in the last 12 months).

Compared to LI, NI were significantly older at the time of initiation of IDU, were significantly less likely to have injected cocaine and significantly more likely to have injected methamphetamines (mainly in Leipzig) in the last 30 days. We did not find any significant differences in unsafe drug injecting behaviour in the last 30 days between LI and NI.

In study cities with syringe vending machines, NI were significantly more likely than LI to have used them to obtain sterile injecting equipment in the last 30 days (53% vs 38%, *p* = 0.006) and to mention them as their main source of sterile syringes and needles (28% vs 16%, *p* = 0.004).

For a detailed comparison of NI and LI see Table 1.

**Table 1:** Sociodemographic characteristics, drug injection behaviour and HCV-status, awareness and testing experience of PWID participating in the German DRUCK-study 2011–2014 by duration of injection drug use

	Injecting drugs < 5 years (*N* = 232)		Injecting drugs ≥ 5 years (*N* = 1827)		*p*
	*n*	Proportion^k^ (%)	*n*	Proportion^k^ (%)	
Sociodemographic characteristics					
Female	73	31.5	403	22.1	0.001^**^
Age ≤ 25 years	71	30.6	62	3.4	< 0.001^***^
2nd-generation migrant^a^	26	11.2	273	14.9	0.128
1st-generation migrant^b^	63	27.2	393	21.5	0.051
Did not graduate from school	46	19.8	250	13.7	0.012*
A-level	23	9.9	182	10.0	0.982
Main place of residence in the last 12 months (max 2 entries)					
Own flat	111	48.1	1040	57.5	0.006^**^
With family or friends	57	24.7	297	16.4	0.002^**^
Homeless, staying in shelters	50	21.7	258	14.3	0.003^**^
Ever homeless^c^	132	57.1	1226	67.3	0.002^**^
Ever in prison	143	61.9	1518	83.3	< 0.001^***^
Released from prison in the last 12 months^d^	37	24.3	332	24.2	0.965
Sources of income in the last 12 months					
Job (including unemployment benefit I)	61	26.4	384	21.2	0.069
State benefits	171	74.0	1548	85.3	< 0.001^***^
Selling newspapers, begging, dealing	110	47.6	673	37.1	0.002^**^
Sex work	17	7.4	60	3.3	0.002^**^
Injection behavior					
Age at first injection < 18 years	19	8.2	623	34.1	< 0.001^***^
Injecting daily in the last 30 days	63	34.2	452	30.1	0.244
Substance injected in the last 30 days^f^					
Heroin	130	56.0	1109	60.8	0.165
Cocaine	73	31.5	752	41.2	0.004^**^
Crack	10	4.3	98	5.4	0.504
Speed (amphetamines)	11	4.7	60	3.3	0.254
Crystal (metamphetamines) ^g^	17	7.4	64	3.5	0.005^**^
Substance consumed in the last 30 days					
Heroin	180	77.6	1355	74.3	0.217
Cocaine	95	41.0	908	49.8	0.011^*^
Crack	54	23.4	461	25.3	0.534
Speed (amphetamines)	49	21.1	234	12.8	0.001^**^
Crystal (metamphetamines)^g^	23	10.0	97	5.3	0.005^**^
Most common setting of drug injection in the last 30 days^h^					
Alone at home^e^	76	42.2	678	45.4	0.425
In consumption room^e,i^	24	27.6	195	31.9	0421
With good acquaintances^e^	75	41.2	484	32.4	0.017^*^
With steady partner^e^	24	13.3	241	16.1	0.317
With hardly known or unknown people^e^	15	8.3	125	8.4	0.984
Unsafe use in the last 30 day^h^					
Used used needles or syringes	19	10.4	133	8.8	0.482
Used water from a shared container	45	24.7	316	21.4	0.302
Used used spoons or filters	40	22.1	280	18.7	0.268
Source for sterile needles and syringes in the last 30 days^h^					
Low threshold services	115	62.2	1069	70.2	0.025^*^
Syringe vending machine^j^	48	52.8	290	37.9	0.006^**^
Pharmacy (bought)	67	38.2	656	44.1	0.142
Access to addiction therapy					
Ever in detoxification	143	61.6	1517	83.2	< 0.001^***^
Ever in weaning/rehabilitation program	80	34.5	1004	55.1	< 0.001^***^
Ever in outpatient substitution therapy	126	54.3	1532	84.0	< 0.001^***^
Currently in outpatient substitution therapy	68	29.3	945	51.8	< 0.001^***^
HCV status, awareness and testing experience					
HCV positive	83	35.8	1270	69.5	< 0.001^***^
Detectable HCV-RNA	63	27.2	836	45.8	< 0.001^***^
Of HCV positive: Unaware of HCV positive status	33	40.7	157	12.6	< 0.001^***^
Ever tested for HCV	153	73.2	1653	93.6	< 0.001^***^
Report negative HCV test, last test > 12 months ago	32	36.8	135	38.8	0.730

^a^Born in Germany, mother and/or father born abroad

^b^Born outside of Germany

^c^Defined as reporting living on the streets or in homeless shelters as main residence in the last 12 months

^d^Not asked in Berlin, Essen

^e^Last 30 days

^f^Substance consumed in last 30 days and most common mode of consumption injection

^g^Methamphethamine use was concentrated in Leipzig (East Germany) and to a lower extent in Munich (South Germany), while it played almost no role in other study cities

^h^Only answered if participants injected drugs in the last 30 days

^i^Information available for Essen, Berlin, Hamburg; reported use of drug consumption rooms varied widely between cities: highest use in Hamburg (> 60% reported by NI and LI), lowest use in Berlin (< 10% reported by NI and LI)

^j^Exist in Berlin, Essen, Cologne, Munich

^k^of responding participants

^*^p < 0.05

^**^p < 0.01

^***^p < 0.001

### HCV-status, history of HCV-testing and awareness of HCV positivity

Of 2077 participating PWID, 66% (*n* = 1361) were HCV-positive: 22% (*n* = 457) were anti-HCV-positive and RNA-negative, 41% (*n* = 857) anti-HCV and RNA-positive, 2.3% (*n* = 47) anti-HCV-negative and RNA-positive. Prevalence of HCV-antibody and/or RNA positivity was 36% in NI and increased with duration of IDU, reaching 72% in participants injecting drugs for 10 years or longer. NI were less likely to be HCV-positive (36% vs 70%, *p* < 0.0001), but among HCV-positives, a higher proportion of NI had detectable HCV-RNA (76% vs 66%, *p* = 0.06); while proportions of NI and LI with chronic infection (anti-HCV-positive, detectable RNA) were comparable (58% vs 63%, *p* = 0.31), the proportions of recent infections (anti-HCV-negative, detectable RNA) were significantly higher in NI (18.1% vs 2.4%, *p* < 0.0001).

HCV positivity among NI was lowest in Leipzig and Munich (both 20%) and highest in Hamburg (58%).

Estimated HCV incidence among NI was 19.6 infections/100 person years at risk (95% CI 16–24); if only participants injecting less than 2 years were considered, estimated incidence was 36.4 infections/100 person years at risk (95% CI 21–56).

NI were less likely to ever have been tested for HCV (73% vs 94%, *p* < 0.0001) and if HCV positive, more likely to be unaware of their HCV status (41% vs 13%, *p* < 0.0001). Reported testing experience among NI was lowest in Leipzig (38%) and in the other study cities ranged between 67% (Cologne) and 89% (Hamburg).

### Uptake of medical care and addiction services: access points used by NI

In order to identify ways to reach NI, this part of the analysis focuses on NI.

Medical care was accessed by 82% of NI (*n* = 192) within the last 12 months. Most commonly mentioned last access points were practices without addiction services (31%, 58/186), practices offering opioid substitution therapy (OST, 30%, 55/186), hospitals (27%, 50/186) and prison hospitals (6.5%, 12/186).

Release from prison in the last 12 months was reported by 24% (37/152 with information, not asked in 2 study cities).

At the time of study, 75% of NI had already received at least one form of addiction therapy: 62% had ever received inpatient detoxification, 54% OST, thereof 29% currently and 34% had ever received long-term addiction therapy (93% as inpatient).

Information on last visit to low threshold drug services was collected in 5 study cities; in those 83% (105/127) reported attendance in the last 30 days.

### Previous HCV testing among NI

Of NI that reported previous HCV-testing, 85% (130/153) provided details on the place where this was performed; the five most commonly mentioned places were practices providing OST (35%, 45/130), hospitals (33%, 43/130), practices without addiction services (14%, 18/130), low threshold drug services (8.5%, 11/130) and prisons (8.5%, 11/130).

Of 56 NI (27%) that reported never having been tested for HCV, 29% (*n* = 16) were HCV-positive. Previous access to addiction services was reported by 57%: 46% had been in inpatient detoxification, 27% in long-term addiction therapy programs and 27% in outpatient OST, thereof 18% currently (see Table 2). At least 21 NI without self-reported HCV testing experience had attended low-threshold drug services in the last 30 days (75%, 21/28 with information).

**Table 2:** HCV-status, awareness, injection behaviour and access to addiction and medical care of new injectors by self-reported HCV-testing experience prior to study

	Reported previous HCV-test (*N* = 153)		Reported no previous HCV-test (*N*= 56)		*p*
	*n*	%^i^	*n*	%^i^	
Sociodemographic characteristic					
Female	49	32.0	14	25.0	0.327
Age 25 years	39	25.5	24	42.9	0.015^*^
1st-generation migrant^a^	45	29.4	12	21.4	0.251
2nd-generation migrant^b^	13	8.5	6	10.7	0.621
Mainly homeless, staying in shelters^c^	31	20.4	17	30.4	0.130
Ever in prison	94	61.8	36	64.3	0.747
Released from prison in the last 12 months^d^	22	23.2	10	24.4	0.876
HCV status					
HCV-positive	64	41.8	16	28.6	0.081
Detectable HCV-RNA	47	30.7	14	25.0	0.421
Of HCV-positive: unaware of HCV-positive status	14	22.6	16	100.0	< 0.001^***^
Access to addiction therapy					
Drug addiction ever treated	128	83.7	32	57.1	< 0.001^***^
Ever in detoxification	105	68.6	26	46.4	0.008^**^
Ever in weaning/rehabilitation program	60	39.2	15	26.8	0.097
Ever in outpatient substitution	101	66.0	15	26.8	< 0.001^***^
Currently in outpatient substitution	52	34.0	10	17.9	0.024^*^
Sought medical care within the last 12 months	127	83.0	44	78.6	0.462
If accessed medical care within 12 months: last access point					
Hospital	25	20.2	17	39.5	0.012^*^
Practice without addiction services	37	29.8	16	37.2	0.371
Practice with OST	44	35.5	6	14.0	0.008^**^
Detention facilities (prison hospital)	11	8.9	1	2.3	0.152
Low threshold drug services	4	3.2	1	2.3	0.765
Rehabilitation	2	1.6	1	2.3	0.762
Local public health office	1	0.8	1	2.3	0.430
Main source for sterile needles and syringes in the last 30 days					
Low threshold services	80	67	21	46	0.011^*^
Bought in pharmacies	21	18	12	26	0.224
Syringe vending machine^e^	16	25	8	36	0.325
Visited low threshold drug services in the last 30 days^f^	77	88	21	75	0.112
Drug injection behaviour in the last 30 days^g^					
Injected drugs	126	82.4	47	83.9	0.789
Daily injection	46	36.8	13	27.7	0.260
Injection of heroin	93	60.8	26	46.4	0.063
Injection of cocaine	55	36.0	13	23.1	0.082
Injection of crack	8	5.3	2	3.6	0.613
Injection of amphetamines	7	4.6	4	7.1	0.462
Injection of methamphetamines^h^	5	3.3	11	19.6	< 0.001

^a^Born in Germany, mother and/or father born abroad

^b^Born outside of Germany

^c^Defined as reporting living on the streets or in homeless shelters as main residence in the last 12 months

^d^Not asked in Berlin, Essen

^e^Substance consumed in the last 30 days and most common mode of consumption injection

^e^Exist in Berlin, Essen, Cologne, Munich

^f^Not asked in Berlin, Essen, Leipzig

^g^Substance consumed in the last 30 days and most common mode of consumption injection

^h^Consumption of methamphethamine was concentrated in Leipzig (East Germany) and to a lower extent in Munich (South Germany), while it played almost no role in other study cities

^i^of responding participants

^*^p < 0.05

^**^p < 0.01

^***^p < 0.001

In the preceding 12 months, 24% (10/41 with information) were released from prison and 79% had sought medical care; most commonly mentioned points of contact were hospitals (40%) and practices without addiction services (37%).

Reported HCV testing experience was higher in females (78% vs 71%, *p* = 0.33), first-generation migrants (29% vs 21%, *p* = 0.25) and NI living in their own accommodation (52% vs 41%, *p* = 0.16); however, differences were not statistically significant (Table 2).

Significantly lower testing experience was reported from NI younger than 25 years (OR in univariable analysis 2.2, 95% CI 1.2–4.2) and those injecting amphetamines or methamphetamines (OR in univariable analysis 4.3, 95% CI 1.8–10.1).

Although low threshold drug services were the most commonly reported source of sterile needles and syringes, NI that denied previous HCV testing were significantly less likely to report them as source (46% vs 67%, *p* = 0.01) and were more likely than NI with testing experience to obtain their syringes and needles from syringe vending machines (36% vs 25%, *p* = 0.3) and pharmacies (26% vs 18%, *p* = 0.2) (Table 2).

NI without OST experience were less likely to ever have undergone HCV testing (56% vs 87%, *p* < 0.0001). They had a shorter duration of IDU (median 2 vs 3 years, *p* = 0.02), a lower HCV prevalence (27% vs 43% with OST, *p* = 0.014) and most commonly accessed medical care in practices without OST (51%), hospitals (26%) and prisons (10%).

HCV-positive NI that last accessed medical care in hospitals were more likely to be unaware of their HCV infection than those that last accessed care in OST-practices (OR 9.9, 95% CI 2.2–43).

## Discussion

We found high HCV positivity and low awareness of HCV-positive status among participating NI. Among NI-estimated HCV incidence was 19.6/100 person years at risk, comparable to the estimated incidence among NI in New York 2000/2001 and slightly lower than in Catalonia 2010/2011 (18 and 25/100 person years at risk, respectively; both using a similar definition of NI, [10, 11]). Estimated HCV incidence was higher in study participants with IDU below 2 years (36/100 person years at risk), supporting that HCV infection often occurs early after initiation of IDU.

HCV prevalence was more than 100-times higher in NI than in a representative study of the “general adult population in Germany” and more than 220-times in LI [4]. Given that seroprevalence increases with time of IDU, it is especially important to reach NI with prevention measures and early HCV-testing.

Studies suggest that awareness of HCV positivity is associated with sustained protective behavioural changes, for example reducing injection risk behaviour [12, 13]. Awareness is a prerequisite for being linked into care and receiving antiviral treatment. Additionally it provides an opportunity for counselling around safer injection practices and linkage to effective prevention measures like OST, needle exchange and other harm reduction services.

In our study, more than 40% of HCV-positive NI were unaware of their HCV status, often with missed opportunities for HCV testing.

More than 50% of NI that reported never having been tested for HCV had previously been in contact with addiction therapy, many in an inpatient setting or in the form of OST, which involves regular engagement with services. Engagement in addiction therapy is an important opportunity for HCV testing that should not be missed.

As could be shown in other studies, we found that NI engaged in OST were more likely to have been tested for HCV than those not receiving OST [14]. However, focusing on OST facilities, does exclude non-opioid dependent PWID and NI that are not (yet) linked to these services.

NI in our study often accessed medical care in hospitals or primary care without focus on addiction care and OST.

In the context of acute medical presentation in hospitals, HCV screening and discussion of test results are challenging. Although an American pilot study showed that emergency room-based HCV testing focused on PWID could be successfully integrated into clinical practice, finding a high prevalence of HCV, the study also encountered significant challenges linking those found to be HCV-positive to care [15]. Nevertheless, testing in emergency departments could at least help improve the level of awareness of one’s HCV-status, a first step in the cascade of care. Opt-out testing for blood borne viruses including HCV reduces barriers and stigma around testing; in several emergency department-based studies, it was feasible and identified unknown HCV-infections [16, 17]. However, implementing routine screening policies in emergency rooms has rarely been attempted in Germany and will face considerable financial and logistical challenges.

Primary medical care is another setting that provides opportunities for HCV-testing. This should be enhanced for example through increasing awareness among physicians and decreasing barriers e.g. through on-site testing [18] or opt-out testing [19].

Low threshold drug services are important needle/syringe exchange sites in Germany. They were frequented by a high proportion of NI making them ideal places for integrated testing. Unfortunately—and in contrast to many other countries—in Germany, it is required that a physician is on-site when HCV-testing is performed and test results are given, which currently greatly limits feasibility for testing in this setting. Training non-physician providers to perform testing could increase feasibility and uptake of HCV-testing and has been successfully employed in other countries e.g. Scotland [20].

Other alternatives might be targeted distribution of HCV self-test kits in low threshold drug services or through vending machines, which would require legal changes (HIV self-tests are currently freely available, but HCV self-tests are not).

In the UK and in the USA, distribution of HIV self-tests through vending machines at venues frequented by gay men is being explored [21, 22]. To our knowledge, this has never been tested for PWID, but since they are used to vending machines for clean injection equipment, it might be worth studying acceptance and use of providing access to HCV self-test kits through vending machines for PWID.

Pharmacies, as the other important supplier of sterile injection equipment, currently play no role in other aspects of the HCV care cascade in Germany. However, studies from other countries suggest that they can be valuable and successfully offer and enhance HCV-testing, linkage to specialist care and even provide treatment [23–25]. Pharmacies could also be a source to access (free or subsidised) HCV self-tests.

In our study, if available, syringe vending machines were an important source for syringes and needles for NI and were more frequently used by NI with shorter duration of IDU.

This finding is in line with a previous study among PWID in Berlin, that users of vending machines often reported a shorter duration of IDU [26]. The authors suggest that in the first time after initiation of IDU, PWID might prefer to obtain their injection equipment anonymously and may not (yet) be willing to visit other drug services [26]. French data showed that vending machines were used by younger PWID, that were hardly reached by other syringe programs [27]. Although they do not facilitate HCV-testing or support NI in other aspects of harm reduction, syringe vending machines are a valuable prevention measure, supplying sterile injection equipment around the clock.

Almost 25% of NI that reported no previous HCV testing had been in prison in the last 12 months. PWID are overrepresented in prison populations worldwide, making prisons suitable settings to deliver HCV prevention (and care) interventions, including HCV-screening [28–30]. According to a review and a cross-sectional survey, measures in European prisons are currently inadequate and need to be scaled up [28, 29]. Universal opt-out HCV-screening in prisons was found to be cost-effective and able to reduce HCV transmission in an American study [31]. It has been introduced in California [32] and has increased screening uptake among prisoners in England [33].

Homelessness was reported by more than 20% of NI in our study, comparable to the findings of a very similar study of NI in Catalonia [10]. Unstable housing has been found to be a risk factor for HCV infection among PWID in Vancouver [34], and in Puerto Rico, homeless PWID were significantly more likely to engage in high-risk injection behaviour than other PWID [35]. There is experience e.g. from London on how to reach the homeless population with HCV services [36, 37] .

## Conclusion

It is important that HCV-counselling and testing are not restricted to medical addiction care, especially for NI. It should be offered in all facilities or settings where NI can be reached, including hospitals and primary medical care, prisons and needle/syringe exchange sites, especially low-threshold drug services. To reach HCV elimination goals and increase feasibility of HCV-testing in the setting of low-threshold drug services which are frequented by the majority of NI, consideration should be given to allow trained non-physician providers to conduct HCV testing. Feasibility and acceptability of HCV self-testing for PWID should be explored.

### Limitations

The number of NI was small, so results have to be interpreted with caution. HCV-testing experience was self-reported; it is therefore possible that participants have been tested without their knowledge or that recall was incorrect. If participants reported no previous HCV-testing, reasons for this were not explored, so we cannot rule out that a test was offered but not accepted. Most seeds (initial study participants selected as recruiters/who “initiate sampling chains”) were recruited through low-threshold drug services which were also used as study sites; this might have led to overestimation of contact with low-threshold drug services in some of the cities. As this was a cross-sectional study, we cannot draw conclusions on causality. There were regional differences in the size and characteristics of the population and as the population of NI is unknown, our sample might not be representative of all new injectors in Germany. Nevertheless the DRUCK study is the first large bio-behavioural study of current PWID in Germany and provides valuable information about characteristics of this group.

## Data Availability

The datasets generated and analysed during the current study are not publicly available to protect research participants’ privacy.

## Abbreviations

HCV: Hepatitis C virus
IDU: Injection drug use
LI: Long-term injectors (injecting drugs for 5 years or more)
NI: New injectors (injecting drugs for less than 5 years)
OST: Opioid substitution therapy
PWID: People who inject drugs
